# Out-of-Pocket Expenditures for Delivery for Maternity Waiting Home Users and Non-users in Rural Zambia

**DOI:** 10.34172/ijhpm.2021.61

**Published:** 2021-06-23

**Authors:** Constance P. Fontanet, Jeanette L. Kaiser, Rachel M. Fong, Thandiwe Ngoma, Jody R. Lori, Godfrey Biemba, Michelle Munro-Kramer, Isaac Sakala, Kathleen Lucile McGlasson, Taryn Vian, Davidson H. Hamer, Peter C. Rockers, Nancy A. Scott

**Affiliations:** ^1^Department of Global Health, Boston University School of Public Health, Boston, MA, USA.; ^2^Department of Research, Right to Care Zambia, Lusaka, Zambia.; ^3^Department of Health Behavior and Biological Sciences, School of Nursing, University of Michigan, Ann Arbor, MI, USA.; ^4^National Health Research Authority, Pediatric Centre of Excellence, Lusaka, Zambia.; ^5^Africare Zambia, Lusaka, Zambia.; ^6^Biostatistics and Epidemiology Data Analytics Center, Boston University School of Public Health, Boston, MA, USA.; ^7^Department of Global Health, School of Nursing and Health Professions, University of San Francisco, San Francisco, CA, USA.; ^8^Section of Infectious Diseases, Boston University School of Medicine, Boston, MA, USA.

**Keywords:** Cost, Skilled Birth Attendance, Obstetric Care, Maternal Health, Zambia, Mothers’ Shelter

## Abstract

**Background:** Utilizing maternity waiting homes (MWHs) is a strategy to improve access to skilled obstetric care in rural Zambia. However, out-of-pocket (OOP) expenses remain a barrier for many women. We assessed delivery-related expenditure for women who used MWHs and those who did not who delivered at a rural health facility.

**Methods:** During the endline of an impact evaluation for an MWH intervention, household surveys (n = 826) were conducted with women who delivered a baby in the previous 13 months at a rural health facility and lived >10 km from a health facility in seven districts of rural Zambia. We captured the amount women reported spending on delivery. We compared OOP spending between women who used MWHs and those who did not. Amounts were converted from Zambian kwacha (ZMW) to US dollar (USD).

**Results:** After controlling for confounders, there was no significant difference in delivery-related expenditure between women who used MWHs (US$40.01) and those who did not (US$36.66) (*P*=.06). Both groups reported baby clothes as the largest expenditure. MWH users reported spending slightly more on accommodation compared to those did not use MWHs, but this difference represents only a fraction of total costs associated with delivery.

**Conclusion:** Findings suggest that for women coming from far away, utilizing MWHs while awaiting delivery is not costlier overall than for women who deliver at a health facility but do not utilize a MWH.

## Background

**Key Messages**

**Implications for policy makers**

- Contrary to popular conceptions, women who use maternity waiting homes (MWHs) in rural Zambia do not report higher out-of-pocket (OOP) spending on delivery-related expenditure compared to women who deliver without using a MWH.
- Interventions leveraging MWHs may not result in negative financial consequences for the women who use them.
- MWHs offer a way to decrease transportation-related barriers to facility-based deliveries without creating additional costs for users.

**Implications for the public**

Pregnant women in low resource settings, such as rural Zambia, face many barriers in accessing adequate maternal health services. Maternity waiting homes (MWHs) are residential dwellings located near qualified health facilities where women can have access to skilled obstetric care. This paper reports the major categories of out-of-pocket (OOP) expenditure for pregnant women who use and who do not use MWHs. Our findings suggest that, in rural Zambia, women who use MWHs before giving birth at a facility do not spend more on delivery-associated expenditure compared to women who do not choose to stay in a MWH. Therefore, using a MWH may not present an added financial burden to low-income pregnant women in rural Zambia.

Maternal health services, including antenatal care (ANC) and delivery at an equipped health facility under the care of a skilled birth attendant, can improve maternal and neonatal health outcomes.^1-4^ Yet, pregnant women in low resource settings, such as rural Zambia, continue to face impediments to accessing these services.^5-9^ Barriers include out-of-pocket (OOP) expenditure for direct and indirect costs, distance, traditional and family influences, and low quality of care, among others.^5-9^

 To address the financial barriers women experience, several sub-Saharan countries implemented policies that eliminate user fees for facility-based delivery services, including delivery.^10^ In 2006, the Zambian government abolished user fees, including those for maternal health services, as part of a similar national effort to increase access to healthcare facilities.^11^ While this policy reduced expenditure for service users, there is mixed evidence regarding the impact of user fee removal on facility-based delivery rates.^11,12^ Additionally, user-fee elimination has not removed all financial barriers. Women across Zambia, Tanzania, and Ethiopia continue to face other financial roadblocks including OOP expenditure for transportation to health facilities, delivery supplies, and baby clothes.^5,7-9,13-16^

 Distance to health facilities is also a well-documented barrier to maternal health service utilization.^6,7,16-18^ This is especially true for women in remote areas, who have trouble finding reliable and affordable transportation to get to a heath facility.^14^ One strategy to address the distance challenge is investing in maternity waiting homes (MWHs), which are residential facilities located near health care facilities qualified to provide basic emergency obstetric care.^18-20^ Although the quality of MWHs varies within and between countries, MWHs are associated with improved maternal health outcomes in several sub-Saharan countries.^21,22^

 MWH use in Zambia has expanded in recent years through the Maternity Homes Alliance (MHA), a collaboration between the Zambian government, two implementing partners, and two academic institutions. The MHA hypothesized that quality MWHs would reduce the distance barrier and increase access to facility-based delivery for rural women in Zambia by bringing pregnant women closer to the health facility as they await delivery.^23^ However, it is possible that reducing one barrier (distance) may exacerbate another (OOP expenditure). Therefore it is essential to examine the financial cost of MWH use to pregnant women living most remotely. Hypothetically, MWHs could decrease transportation costs because of the opportunity to plan for transport and avoid paying a premium on urgent transport. Conversely, MWHs could increase accommodation-related costs if pregnant women are charged for their MWH stay or women using them encounter other unanticipated costs. Yet, there is little empirical evidence on whether expenditure differences exist between those who use MWHs and those who do not (referred throughout the rest of this paper as ‘users’ and ‘non-users’ respectively).^24^

 Within a broader impact evaluation of the MHA, we analyzed self-reported OOP expenditure for women living remotely who recently delivered at a health facility. We compared OOP spending of women who stayed at MWHs and those who did not for their most recent delivery to better understand how MWH use factors into OOP expenditure for facility delivery among socioeconomically disadvantaged women in rural Zambia.

## Methods

###  Study Setting

 Zambia is divided in ten provinces. The MHA project is based in the following rural districts in three provinces; Choma, Kalomo, and Pemba in Southern Province, Nyimba and Lundazi in Eastern Province and Mansa and Chembe in Luapula province.^23^ These districts are primarily rural, poor, and have low utilization of maternal health services.^25,26^

###  Intervention Description

 The study team conducted formative research with community stakeholders to determine an acceptable, feasible, and sustainable MWH intervention for the relevant districts.^27-29^ MWHs were intended to be utilized by expecting mothers within 1-2 weeks of delivery, with a focus on the most remote women, defined as living >10 km from a health facility that provides maternal health services. MWHs were designed to be comfortable, safe, and culturally appropriate; and to be linked with the health system to ensure pregnant women receive clinical services.^23,27-29^ The MWHs provided cooking space and utensils but did not provide food for women. Consistent with government policy, the MWHs were designed to be free of charge for women and their companions awaiting ANC, labor and delivery, or postnatal care.

###  Study Design and Data Collection 

 The overarching study has been described elsewhere.^23^ In 2018, following the implementation of the MWH intervention (endline), we conducted a cross-sectional household survey with a random sample of women who delivered a baby in the 13 months prior to the survey and who lived more than 10 km from the 20 rural health center study sites that received the MWH intervention. The household survey captured basic demographic information for the household and the woman, and information about the woman’s most recent delivery, delivery location, MWH use, and expenditures associated with delivery. Expenditure was reported in the local currency, the Zambian kwacha (ZMW). Women were identified using a multistage random sampling procedure. The first sampling stage included visiting every village within the catchment area of each study site and informing the local village leader of the goal of the survey. Using GPS coordinates, we calculated the distance between health facilities and villages. We then developed a sampling frame for all villages within each catchment area located further than 10 km (rounding up from 9.5 km) from the health facility. Next, we randomly selected a sample of 10 villages from each catchment area with probability proportional to population size using a random number generator. Finally, women who had a delivery in the last year in the households within the selected villages were identified. Further details about our sampling strategy have been published elsewhere.^23^

 For this analysis, we excluded women who reported not knowing whether they had used a MWH (n = 1) and those who reported having used the MWH for a reason other than awaiting delivery, such as ANC visits (n = 26). We also excluded respondents (n = 2) who reported not knowing whether they had saved money for delivery. Additionally, we excluded women who delivered at home (n = 82), on the way to a facility (n = 25), or at a hospital (n = 251). Lastly, we excluded 2 users and 2 non-users who reported spending more than US$100 in any category besides total expenditure, as this spending behavior was considered an extreme outlier and these records may have resulted from incorrect data entry, such as using Malawian kwacha rather than ZMW. Our final analytic sample included 826 women who delivered at a rural health center within the intervention sites.

###  Data Management and Analysis

 For this analysis, we defined OOP expenditure as direct non-medical costs to patients themselves or to their relatives,^30^ as these costs would have been most likely to affect the financial decision of using a MWH in the target population. We examined expenditure data from four categories: (1) delivery supplies such as disinfectant, gloves, cord clamps, or a plastic sheet; (2) baby clothes/blanket; (3) transport to and from the delivery location; and (4) accommodation while awaiting delivery. We defined accommodation as expenses incurred for staying at a MWH or another type of accommodation before delivery at a health facility. We did not explicitly inquire about food-related expenses, which may have been reported under the “accommodation” category even though food was not provided by all the MWHs. Each site decided whether to provide food, and did not always do so consistently. We calculated total expenditure based on the sum in expenditure of these categories. We collected other categories of expenditure that are not analyzed here. Expenditure data were converted to US dollars (USD) using the monthly average ZMW to USD exchange rates from September 2017 to October 2018 (10 ZMW equaling US$1).^31^

 We created the following variables for this analysis: whether a respondent reported spending anything on delivery overall or within each expenditure category, season of delivery (rainy or dry), and wealth quartiles. Dry season was defined as the period from May to November and rainy season from December to April. Wealth quartiles were calculated based on responses to a series of household asset questions.^25^ The district of Choma was combined with the district of Pemba as were Mansa and Chembe districts, as each pair of districts is demographically similar and they had been previously unified administratively.

 We calculated descriptive statistics for MWH users and non-users and compared the two groups using chi-square tests of homogeneity and two-sample *t *tests, while accounting for clustering at the health facility catchment area level.

 We calculated the proportion of women who reported spending anything in each of the expenditure categories. We then calculated the mean and standard deviation (SD) for the reported total and by category expenditure of the total sample, which included individuals who did not report any expenditure. We repeated the calculations for only those who had spent money within each of the categories. We display boxplots to show distribution for the five categories of expenditure between users and non-users in the intervention group.

 To adjust for differences between users and non-users, we employed a two-part modelling approach to examine between-group differences in mean total expenditure (see Supplementary file 1 for equations). This approach is widely used to measure health care expenditures.^32^ As there were few statistically significant between-group differences in our sample, we only included ANC attendance (four or more visits) and whether a woman saved for delivery as covariates. Because users and non-users were evenly distributed among different districts, we did not include districts as covariates in our model. For the 1st part of the model, we used a logit model to predict the likelihood of any spending.For the 2nd part of the model, among those had had non-zero total expenditure, we used generalized linear models. Among those who reported non-zero expenditure, we fit a log transformed generalized linear model to examine the association between select covariates and level of expenditure. All data cleaning and analysis were conducted using SAS version 9.4 (SAS Institute, Cary, NC, USA).

## Results

###  Sample Characteristics

 A sample of 826 women were included in this analysis, of which 543 (65.7%) reported staying at the MWH while awaiting delivery (MWH users). Households were generally poor, reporting high rates of non-improved toilets (81.8%) and no electricity (98.8%). Households were located a median of 12.5 km from their assigned primary health centers. Respondents had a median age of 24 years and 7 years of education. The majority were married or cohabitating (89.3%). Just under 20% of women were reporting their first pregnancy; about 72% had attended the recommended 4 or more ANC visits. The majority of respondents reported having set aside money for delivery (77.5%). Both groups reported similar mean recall periods, defined as the length of time between giving birth and completing the questionnaire (6.7 months for users and 6.9 months for non-users).

 We found few demographic differences between MWH users and non-users (Table 1). Additionally, users were more likely to report having set money aside for delivery (*P* =.001) and to have completed 4 or more ANC visits (*P* =.01).

**Table 1:** Intervention Sample Characteristics (n = 826)

**Household-Level Characteristics**	**MWH Users ** **(n = 543)**	**MWH Non-users (n = 283)**	**Woman-Level Characteristics**	**MWH Users ** **(n = 543)**	**MWH Non-users (n = 283)**
**Non-improved water source** ^a^, No. (%)	284 (52.3)	149 (52.7)	**Age**, No. (%)		
**Non-improved toilet** ^b^, No. (%)	447 (82.3)	229 (80.9)	15-19	83 (15.5)	44 (15.8)
**No electricity**, No. (%)	537 (98.9)	279 (98.6)	20-24	174 (32.6)	107 (38.4)
**House has earth or sand floors**, No. (%)	457 (84.2)	240 (84.8)	25-34	202 (37.8)	85 (30.5)
**Charcoal or wood cooking fuel**, No. (%)	167 (30.8)	95 (33.6)	35+	75 (14.0)	43 (15.4)
**Total household members**, median (IQR)	6 (4-8)	6 (4-8)	Median (IQR)	25 (21-32)	24 (21-30)
**Wealth quartile**, No. (%)			**Years of education**, median (IQR)	7 (4-8)	7 (4-8)
1 (lowest)	120 (22.2)	71 (25.1)	**Marital status**, No. (%)		
2	142 (26.3)	70 (24.7)	Never married	31 (5.8)	23 (8.2)
3	132 (24.4)	75 (26.5)	Divorced/separated or widowed	24 (4.5)	9 (3.2)
4 (highest)	147 (27.2)	67 (23.7)	Married/cohabitating	479 (89.7)	249 (88.6)
**Distance from village center to health facility (km), **median (IQR)	12.5 (11.0-16.0)	12.7 (11.1-16.8)	**Gravida**, median (IQR)	3 (2-5)	3 (2-5)
**District**, No. (%)			**Parity**, median (IQR)	3 (1-5)	3 (2-5)
Kalomo	134 (24.7)	59 (20.8)	**Primigravida (first pregnancy)**, No. (%)	442 (81.6)	227 (80.2)
Choma/Pemba	116 (21.4)	82 (29.0)	**Completed 4 or more ANC visits**, No. (%)*	411 (75.7)	187 (66.1)
Lundazi	148 (27.3)	69 (24.4)	**Saved for delivery**, No. (%)*	439 (80.9)	201 (71.0)
Nyimba	22 (4.0)	5 (1.8)	**Dry season**, No. (%)	327 (60.2)	181 (64.0)
Mansa/Chembe	123 (22.7)	68 (24.0)	**Motorized transport**, No. (%)	181 (34.0)	110 (39.3)

Abbreviations: MWH, maternity waiting home; IQR, interquartile range; ANC, antenatal care. ^a^Non-improved water sources do not properly protect water from contamination. ^b^Non-improved toilets do not properly separate human excreta from human contact. * Significant at.05 level.

###  Expenditure Among the Total Sample

 Respondents reported spending a mean of US$38.93 (SD: US$23.56) on delivery-related expenses. Nearly all respondents (96.6%) reported spending some amount of money for their delivery. On average, baby clothes and delivery supplies comprised 65% and 25%, respectively, of total expenditure. The majority of both MWH users and non-users reported spending in these categories (Figure). In comparison, transportation costs were a moderate contributor to total expenditure, with about a third of the sample reporting having spent money on transport (Figure). Accommodation-related expenditure was less frequent, with 17.0% of the total sample reporting having spent money in that category.

**Figure:**
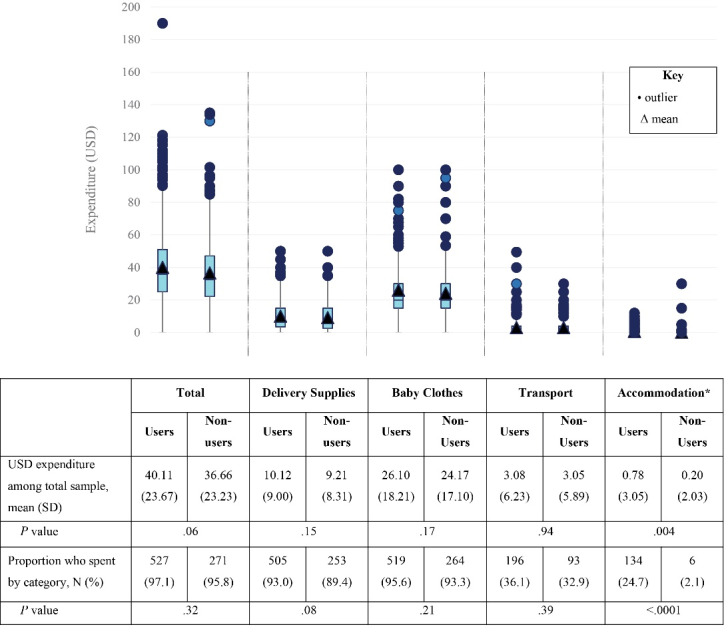
Distribution of Expenditure by Expenditure Categories in MWH Users and Non-users. Abbreviations: MWH, maternity waiting home; SD: standard deviation; USD, US dollars.

###  Expenditure Among Spenders

 Users and non-users were as likely to have spent any money on delivery (*P* =.32). Only accommodation-related expenses showed a difference in user vs. non-user spending, with users being more likely to spend money (Figure, *P* < .001).

 Looking only at those who had reported some delivery-associated spending, we found no significant differences in mean expenditure between user groups. This was true for total expenditure and expenditure by category (Table 2). Among those who had spent any money on accommodation, mean accommodation-related expenditure was not different between users and non-users (Table 2).

**Table 2:** Mean Expenditure Among Spenders in Users and Non-users

	**Mean Spending Among Spenders (SD)**		
	**MWH Users**	**MWH Non-users**	***P*** ** Value**
Total	41.33 (22.95)	38.29 (22.39)	.11
Delivery supplies	10.88 (8.87)	10.30 (8.12)	.36
Clothes	27.30 (17.71)	25.91 (16.38)	.33
Transport	8.54 (7.81)	9.29 (6.91)	.40
Accommodation	3.14 (5.51)	9.45 (11.33)	.15

Abbreviations: MWH, maternity waiting home; SD, standard deviation.

###  Adjusted Analysis

 We found differences between users and non-users related to having saved for delivery and having received 4 or more ANC visits. Notably, having saved for delivery was significantly associated with having spent any money on delivery (*P* =.04).

 Women who had saved for delivery had 2.53 times the odds (*P* =.02) of spending anything on delivery-related costs and spent US$10.16 (*P* <.0001) more on delivery-related costs compared to women who did not report having saved for delivery after controlling for all other predictors in the model (Table 3). MWH utilization and attendance of 4 or more ANC visits were not significantly associated with total expenditure in these models. After adjusting for having saved for delivery, there were no differences between users and non-users in terms of odds of having spent any money and amount spent among spenders (Table 3).

**Table 3:** Predictors of Any Expenditure and Total Expenditure for Delivery Among Spenders

	**Predictors of any Expenditure (Total Expenditure >0) **		**Predictors of Expenditure Amount Among Spenders**		
	**Adjusted Odds Ratio (95% CI)**	***P*** ** Value**	**Adjusted Coefficient (95% CI)**	**Adjusted Effect (USD) (95% CI)**	***P*** ** Value**
MWH Use	1.25 (0.57, 2.71)	0.58	0.05 (-0.04, 0.14)	1.55 (-1.18, 4.54)	.31
ANC visits	1.58 (0.72, 3.46)	0.26	0.03 (-0.05, 0.11)	0.92 (-1.47, 3.51)	.47
Saved for delivery	2.53 (1.16, 5.49)	0.02	0.29 (0.18, 0.39)	10.16 (5.95, 14.40)	<.0001
			Intercept	5.71	
			*P* value	<.0001	

Abbreviations: MWH, maternity waiting home; ANC, antenatal care.

## Discussion

 We examined the OOP expenditures of pregnant women to determine whether known barriers to facility-based deliveries – such as transportation costs – are reduced, and to explore whether other financial barriers change or appear in their place. We found no significant differences in OOP expenditures whether the pregnant woman chose to stay in a MWH while awaiting delivery or not.

 Costs of baby clothes or *chitenge* (Zambian fabric) for delivery had previously been reported as a large contributor to OOP spending.^13-15^ Specifically, we had previously found that the expectation that women will bring their own baby clothes represents an additional barrier to facility-based delivery.^13^ In this analysis, baby clothes continued to make up the majority of OOP expenditure in both groups. The cost of baby clothes may remain a financial barrier to facility-based delivery, but does not differ between MWH users and non-users and is therefore unlikely to have influenced a pregnant woman’s decision to use a MWH.

 Transportation costs represented a small portion of total expenditure in this population and spending on transportation was not different between groups. Additionally, those who reported high transport costs had used motorized transport, among both users and non-users. During a previous analysis of expenditure associated with delivery among pregnant women in rural Zambia,^13^ we found that transportation-related expenditure contributed only marginally to total expenditure. Recent qualitative literature suggests women often perceive that transportation, and particularly the cost of transportation, is a barrier to facility delivery.^14,15,33^ Users and non-users in this study reported similar levels of transportation-related expenditure, suggesting that using a MWH does not lead to decreased transportation costs for pregnant women, but does not increase them either. While finding reliable transportation might remain a barrier to access to a health facility, transportation cost may not be as important as previous qualitative studies have suggested.^13,14,16^ Further research should explore the availability and the cost of transportation as barriers to facility-based delivery.

 Additionally, we found that women utilizing the MWHs expend significantly more on accommodation than those who do not. While the MWHs were intended to be free of charge, some MWHs reportedly charged a small fee to women at various points in time, for expenses like maintenance. This likely accounts for the differences observed in expenditures related to accommodation between users and non-users. Moreover, because we do not know how respondents defined accommodation spending, it is possible that women included items needed during the MWH stay, such as soap or food, in that expenditure category. Food in particular has previously been reported as a contributor to delivery-related costs^34^ and a determinant in willingness to use MWHs,^35,36^ and may have impacted users more heavily than non-users. As such, food- and other accommodation-related costs merit further exploration. However, accommodation-related spending was small proportionate to other costs and there were no differences between users and non-users in mean accommodation-related expenditure among spenders. This suggests that while MWH users are more likely to spend money on accommodation than non-users, the actual amount spent is similar in both groups and may be negligible when considering total expenditure.

 Finally, we found that the total amount women expend for facility-based delivery after staying at a MWH was not significantly different from that of non-users. Additionally, there were no differences in total expenditure between users and non-users after adjusting for having saved for delivery. We believe that this is because some of the women who decided early in their pregnancy to stay at the MWH started saving money in order to meet this goal, rather than because women in the MWH user group were more inclined to save for delivery regardless of their intent to use a MWH. Saving for delivery has previously been associated with both higher overall expenditure on delivery and higher likelihood of delivering at a facility.^37^ The effect of saving for delivery warrants further exploration, as having more money to spend may allow women to purchase the necessary supplies for delivery, and has been previously reported to be associated with better treatment by staff at health facilities.^37^ Interventions that target women’s ability to adequately save for delivery may help reduce financial barriers to facility-based delivery, regardless of MWH use.

 After a review of the current literature, we found only one study comparing OOP expenditure between MWH users and non-users.^24^ This study was conducted among Ethiopian MWH users and non-users who had delivered at hospitals and health centers rather than only primary health centers. Expenditure categories were defined differently, limiting the comparability of our results. Additionally, the authors found major differences between MWH users and non-users, whereas our analysis showed very few.^24^ They concluded that MWH users have significantly higher OOP costs than non-users, but we believe this difference emerged from different sampling frames and methodology.^24^

 Overall, our findings suggest that, for women living most remotely who deliver at rural health facilities in Zambia, using a MWH does not cost significantly more than delivering at a rural health facility without using a MWH when considering key categories of common delivery-related expenses. The use of MWHs is unlikely to impose additional financial burden on rural Zambian women.

###  Strengths and Limitations

 Our study benefits from a large sample size and the measurement of the delivery-associated expenditure which have consistently been reported as a barrier to facility delivery and MWH use. Additionally, to our knowledge, this is the first comparison of expenditure between MWH users and non-users in rural Zambia and only the second comparison of expenditure between MWH users and non-users overall.^24^ Understanding what is actually driving OOP costs could help program managers to better design interventions to increase access to maternal health services and improve health outcomes. However, our analysis has some limitations. First, we focused our analysis on women who delivered at a rural health center. This limits our ability to generalize our results to pregnancies requiring referral to a more specialized facility. However, our findings apply to the majority of deliveries. Second, we did not collect quantitative information on food-associated or opportunity costs, which may limit our understanding of total spending associated with delivery. Additionally, we did not collect qualitative information on what constituted ‘accommodation-related’ expenses, which may limit our understanding of why pregnant women spend money when staying at a MWH. We also did not assess our sample’s ability to pay. However, since the wealth distributions were similar between users and non-users, it is unlikely that the two groups had a differential ability to pay. To comprehensively assess the financial impact of MWHs, future studies should include more information about these costs, as well as those incurred by other family members and the health system. Further studies should also explore willingness and ability to pay for OOP expenditures to better understand implications on the target population’s financial situation. Third, our information was self-reported and may be subject to recall bias as some of these data were collected up to one year after delivery. However, it is unlikely that recall differed between users and non-users, especially as mean recall time was similar in both groups. Finally, our point estimates are limited by potential fluctuations in the exchange rate between the Zambian kwacha and US dollar during the study.

## Conclusion

 We found that staying at a MWH was not associated with higher delivery-associated OOP costs. While the difference in accommodation costs was statistically significant between MWH users and non-users, this difference represented a small fraction of the total costs incurred by women. Slight differences in total expenditure may be driven by whether women save for delivery. Our findings suggest that OOP costs may not present as much of a barrier to MWH use and facility delivery as previously assumed.

## Acknowledgements

 The authors would like to thank the Zambian Ministry of Health at the National, Provincial, and District levels, as well as the traditional leadership of the relevant areas, for their approval and support. We are deeply grateful for our respondents, who participated in the household survey and shared their time and experiences. We appreciate the assistance of community volunteers for their introductions to the study villages and households. We also wish to thank Elizabeth G. Henry, who played an essential role in data management. Lastly, we would like to thank the data collectors and study staff for their diligent work and constant efforts.

## Ethical issues

 The appropriate Institutional Review Board (IRBs) granted ethical approval for the impact evaluation.^23^ Additionally, permission was granted by the National Health Research Authority, the Ministry of Health at the national, provincial, and district levels, and the traditional leaders overseeing the study areas. All respondents provided written informed consent through signature or a thumbprint. Women between 15 and 17 years of age provided written informed assent and written informed consent was obtained from their guardian. Prior to data collection, a team of local data collectors was trained in research ethics and data collection methods. The overarching evaluation was registered under Clinicaltrials.gov (identifier: NCT 02620436).

## Competing interests

 All authors except CPF report grants from MSD for Mothers, the Bill & Melinda Gates Foundation, and the ELMA Foundation during the conduct of the study.

## Authors’ contributions

 Conception and design: CPF, JLK, RMF, NAS, and PR. Acquisition of data: JLK, TN, RMF, and KLM. Analysis and interpretation of data: CPF, JLK, RMF, NAS, and PR. Drafting of the manuscript: CPF, JLK, RMF, and NAS. Critical revision of the manuscript for important intellectual content: CPF, JLK, RMF, TN, JRL, GB, MNK, IS, KLM, TV, DHH, PR, and NAS. Statistical analysis: CPF, JLK, RMF, NA, and PR. Obtaining funding: NAS and JRL. Administrative, technical, or material support: TN, GB, MMK, and IS. Supervision: NAS and PR.

## Funding

 This work was supported by, developed, and implemented in collaboration with *MSD for Mothers*, MSD’s 10-year, $500 million initiative to help create a world where no woman dies giving life. *MSD for Mothers* is an initiative of Merck & Co., Inc., Kenilworth, NJ, USA (MRK 1846-06500.COL). The development of this article was additionally supported in part by the Bill & Melinda Gates Foundation (OPP1130329 and OPP1130334) and The ELMA Foundation (ELMA-15-F0017 and ELMA-15-F0010). The funders had no role in study design, data collection and analysis, decision to publish, or preparation of the manuscript. The content is solely the responsibility of the authors and does not reflect positions or policies of MSD, the Bill & Melinda Gates Foundation, or The ELMA Foundation.

## Supplementary files

Supplementary file 1.Two-Part Model Equations.

## References

[1] Campbell OM, Graham WJ (2006). Strategies for reducing maternal mortality: getting on with what works. Lancet.

[2] World Health Organization (WHO). Postnatal Care of the Mother and Newborn 2013. Geneva, Switzerland; 2013. 24624481

[3] World Health Organization (WHO). Guidelines on Maternal, Newborn, Child and Adolescent Health. WHO; 2014.

[4] World Health Organization (WHO). WHO Recommendations on Antenatal Care for a Positive Pregnancy Experience. WHO; 2016. 28079998

[5] Bohren MA, Hunter EC, Munthe-Kaas HM, Souza JP, Vogel JP, Gülmezoglu AM (2014). Facilitators and barriers to facility-based delivery in low- and middle-income countries: a qualitative evidence synthesis. Reprod Health.

[6] Moyer CA, Mustafa A (2013). Drivers and deterrents of facility delivery in sub-Saharan Africa: a systematic review. Reprod Health.

[7] Gabrysch S, Campbell OM (2009). Still too far to walk: literature review of the determinants of delivery service use. BMC Pregnancy Childbirth.

[8] Kyei-Nimakoh M, Carolan-Olah M, McCann TV (2017). Access barriers to obstetric care at health facilities in sub-Saharan Africa-a systematic review. Syst Rev.

[9] Kaiser JL, Fong RM, Hamer DH (2019). How a woman’s interpersonal relationships can delay care-seeking and access during the maternity period in rural Zambia: an intersection of the Social Ecological Model with the Three Delays Framework. Soc Sci Med.

[10] McPake B, Witter S, Ensor T (2013). Removing financial barriers to access reproductive, maternal and newborn health services: the challenges and policy implications for human resources for health. Hum Resour Health.

[11] Masiye F, Kaonga O, Kirigia JM (2016). Does user fee removal policy provide financial protection from catastrophic health care payments? evidence from Zambia. PLoS One.

[12] Lépine A, Lagarde M, Le Nestour A (2018). How effective and fair is user fee removal? evidence from Zambia using a pooled synthetic control. Health Econ.

[13] Kaiser JL, McGlasson KL, Rockers PC (2019). Out-of-pocket expenditure for home and facility-based delivery among rural women in Zambia: a mixed-methods, cross-sectional study. Int J Womens Health.

[14] Scott NA, Henry EG, Kaiser JL (2018). Factors affecting home delivery among women living in remote areas of rural Zambia: a cross-sectional, mixed-methods analysis. Int J Womens Health.

[15] Sialubanje C, Massar K, Hamer DH, Ruiter RA (2015). Reasons for home delivery and use of traditional birth attendants in rural Zambia: a qualitative study. BMC Pregnancy Childbirth.

[16] Sacks E, Vail D, Austin-Evelyn K (2016). Factors influencing modes of transport and travel time for obstetric care: a mixed methods study in Zambia and Uganda. Health Policy Plan.

[17] Nesbitt RC, Lohela TJ, Soremekun S (2016). The influence of distance and quality of care on place of delivery in rural Ghana. Sci Rep.

[18] Sialubanje C, Massar K, Hamer DH, Ruiter RA (2014). Understanding the psychosocial and environmental factors and barriers affecting utilization of maternal healthcare services in Kalomo, Zambia: a qualitative study. Health Educ Res.

[19] van Lonkhuijzen L, Stekelenburg J, van Roosmalen J (2012). Maternity waiting facilities for improving maternal and neonatal outcome in low-resource countries. Cochrane Database Syst Rev.

[20] Chama-Chiliba CM, Koch SF (2016). An assessment of the effect of user fee policy reform on facility-based deliveries in rural Zambia. BMC Res Notes.

[21] Gaym A, Pearson L, Soe KW (2012). Maternity waiting homes in Ethiopia--three decades experience. Ethiop Med J.

[22] Henry EG, Semrau K, Hamer DH (2017). The influence of quality maternity waiting homes on utilization of facilities for delivery in rural Zambia. Reprod Health.

[23] Scott NA, Kaiser JL, Vian T (2018). Impact of maternity waiting homes on facility delivery among remote households in Zambia: protocol for a quasiexperimental, mixed-methods study. BMJ Open.

[24] Getachew B, Liabsuetrakul T (2019). Health care expenditure for delivery care between maternity waiting home users and nonusers in Ethiopia. Int J Health Plann Manage.

[25] Central Statistical Office (CSO) [Zambia], Ministry of Health (MOH) [Zambia], ICF International. Zambia Demographic and Health Survey 2013-14. Rockville, Maryland, USA: CSO, MOH, ICF International; 2015.

[26] Republic of Zambia Ministry of Health. The 2012 List of Health Facilities in Zambia. Ministry of Health; 2013.

[27] Lori JR, Munro-Kramer ML, Mdluli EA, Musonda Mrs GK, Boyd CJ (2016). Developing a community driven sustainable model of maternity waiting homes for rural Zambia. Midwifery.

[28] Scott NA, Vian T, Kaiser JL (2018). Listening to the community: using formative research to strengthen maternity waiting homes in Zambia. PLoS One.

[29] Chibuye PS, Bazant ES, Wallon M, Rao N, Fruhauf T (2018). Experiences with and expectations of maternity waiting homes in Luapula province, Zambia: a mixed-methods, cross-sectional study with women, community groups and stakeholders. BMC Pregnancy Childbirth.

[30] Kobelt G. Cost Data for Economic Evaluation. In: Health Economics: An Introduction to Economic Evaluation. 3rd ed. Office of Health Economics; 2013.

[31] Bank of Zambia. Historical Average Exchange Rates Series. http://www.boz.zm/average-exchange-rates.htm. Accessed July 18, 2018.

[32] Deb P, Norton EC (2018). Modeling health care expenditures and use. Annu Rev Public Health.

[33] Ng’anjo Phiri S, Fylkesnes K, Ruano AL, Moland KM (2014). ‘Born before arrival’: user and provider perspectives on health facility childbirths in Kapiri Mposhi district, Zambia. BMC Pregnancy Childbirth.

[34] Perkins M, Brazier E, Themmen E (2009). Out-of-pocket costs for facility-based maternity care in three African countries. Health Policy Plan.

[35] Vian T, White EE, Biemba G, Mataka K, Scott N (2017). Willingness to pay for a maternity waiting home stay in Zambia. J Midwifery Womens Health.

[36] Sialubanje C, Massar K, van der Pijl MS, Kirch EM, Hamer DH, Ruiter RA (2015). Improving access to skilled facility-based delivery services: Women’s beliefs on facilitators and barriers to the utilisation of maternity waiting homes in rural Zambia. Reprod Health.

[37] Chiu C, Scott NA, Kaiser JL (2019). Household saving during pregnancy and facility delivery in Zambia: a cross-sectional study. Health Policy Plan.

