# Supplementary material for: Out-of-Pocket Expenditures for Delivery for Maternity Waiting Home Users and Non-users in Rural Zambia
Source: Int J Health Policy Manag. 2021 Jun 23;11(8):1542–9. doi: 10.34172/ijhpm.2021.61 (PMC9808339; doi:10.34172/ijhpm.2021.61)
Supplement: Supplementary file 1 — Two-Part Model Equations. [file ijhpm-11-1542-s001.pdf]

**Article title:** Out-of-Pocket Expenditures for Delivery for Maternity Waiting Home Users and Non-users in Rural Zambia

**Journal name:** International Journal of Health Policy and Management (IJHPM)

**Authors' information**: Constance P. Fontanet[1]*, Jeanette L. Kaiser[1], Rachel M. Fong[1], Thandiwe Ngoma[2], Jody R. Lori[3], Godfrey Biemba[4], Isaac Sakala[5], Kathleen Lucile McGlasson[6], Taryn Vian[7], Davidson H. Hamer[8,9], Peter C. Rockers[1], Nancy A. Scott[1]

[1]Department of Global Health, Boston University School of Public Health, Boston, MA, USA.

[2]Department of Research, Right to Care Zambia, Lusaka, Zambia.

[3]Department of Health Behavior and Biological Sciences, School of Nursing, University of Michigan, Ann Arbor, MI, USA.

[4]National Health Research Authority, Pediatric Centre of Excellence, Lusaka, Zambia.

[5]Africare Zambia, Lusaka, Zambia.

[6]Biostatistics and Epidemiology Data Analytics Center, Boston University School of Public Health, Boston, MA, USA.

[7]Department of Global Health, School of Nursing and Health Professions, University of San Francisco, San Francisco, CA, USA.

[8]Department of Global Health, Boston University School of Public Health, Boston, MA, USA.

[9]Section of Infectious Diseases, Boston University School of Medicine, Boston, MA, USA.

(*Corresponding author: fontanet@bu.edu)

**Supplementary file 1.** Two-Part Model Equations

Part 1

$$(1) \quad Spending_i=\alpha+\eta 1(MWHuse_i)+\eta 2(Savings_i)+ \eta 2(ANCvisits_i)$$

where $Spending_i$ it is the savings outcome for individual i, $MWHuse_i$ indicates whether individual i used a MWH, $Savings_i$ indicates whether individual i reported having saved for delivery, and $ANCvisits_i$ indicates whether individual i had attended 4 or more ANC visits). The coefficient of interest is η1.

Part 2

$$(2) \qquad Amount_i = \alpha + \eta 1(MWHuse_i) + \eta 2(Savings_i) + \eta 2(ANCvisits_i)$$

where $Amount_i$ is the amount of spending outcome for individual i, $MWHuse_i$ indicates whether individual i used a MWH, $Savings_i$ indicates whether individual i reported having saved for delivery, and $ANCvisits_i$ indicates whether individual i had attended 4 or more ANC visits). The coefficient of interest is $\eta 1$.
